# Bromo­triphenyl­silane

**DOI:** 10.1107/S1600536808010520

**Published:** 2008-04-23

**Authors:** Hannah Steinert, Hans-Wolfram Lerner, Michael Bolte

**Affiliations:** a Institut für Anorganische Chemie, J. W. Goethe-Universität Frankfurt, Max-von-Laue-Strasse 7, 60438 Frankfurt/Main, Germany

## Abstract

The title compound, C_18_H_15_BrSi, crystallizes with two almost identical mol­ecules (r.m.s. deviation for all non-H atoms = 0.074 Å) in the asymmetric unit. It is isomorphous with chloro­triphenyl­silane.

## Related literature

For related literature, see: Lerner *et al.* (2001 ▶, 2005 ▶, 2006 ▶); Lobkovskii *et al.* (1981 ▶).
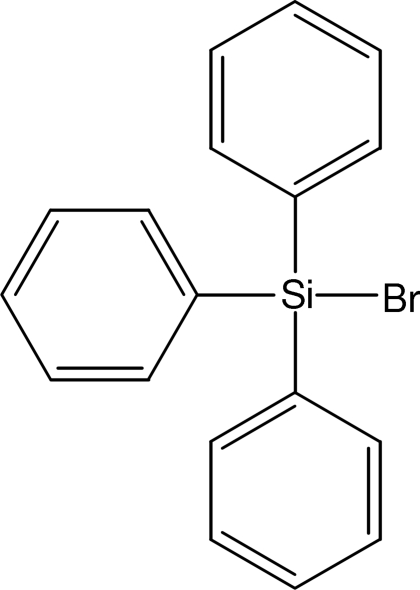


## Experimental

### 

#### Crystal data

C_18_H_15_BrSi
*M* *_r_* = 339.30
Monoclinic, 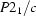

*a* = 18.6306 (13) Å
*b* = 9.6160 (4) Å
*c* = 18.3618 (13) Å
β = 107.174 (5)°
*V* = 3142.9 (3) Å^3^
*Z* = 8
Mo *K*α radiation
μ = 2.68 mm^−1^
*T* = 173 (2) K
0.31 × 0.25 × 0.19 mm

#### Data collection

Stoe IPDSII two-circle diffractometer
Absorption correction: multi-scan (*MULABS*; Spek, 2003 ▶; Blessing, 1995 ▶) *T*_min_ = 0.490, *T*_max_ = 0.630
47324 measured reflections
6141 independent reflections
4739 reflections with *I* > 2σ(*I*)
*R*_int_ = 0.073

#### Refinement

*R*[*F* ^2^ > 2σ(*F* ^2^)] = 0.094
*wR*(*F* ^2^) = 0.288
*S* = 1.16
6141 reflections
362 parameters
H-atom parameters constrained
Δρ_max_ = 2.34 e Å^−3^
Δρ_min_ = −2.22 e Å^−3^

### 

Data collection: *X-AREA* (Stoe & Cie, 2001 ▶); cell refinement: *X-AREA*; data reduction: *X-AREA*; program(s) used to solve structure: *SHELXS97* (Sheldrick, 2008 ▶); program(s) used to refine structure: *SHELXL97* (Sheldrick, 2008 ▶); molecular graphics: *XP* in *SHELXTL-Plus* (Sheldrick, 2008 ▶); software used to prepare material for publication: *PLATON* (Spek, 2003 ▶).

## Supplementary Material

Crystal structure: contains datablocks I, global. DOI: 10.1107/S1600536808010520/pv2078sup1.cif

Structure factors: contains datablocks I. DOI: 10.1107/S1600536808010520/pv2078Isup2.hkl

Additional supplementary materials:  crystallographic information; 3D view; checkCIF report

## Figures and Tables

**Fig. 1.**
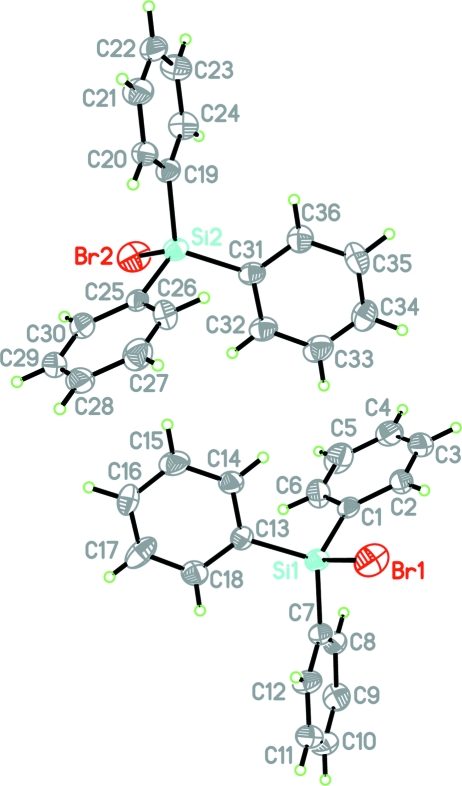
Perspective view of the title compound with the atom numbering scheme; displacement ellipsoids are at the 50% probability level; H atoms are drawn as small spheres of arbitrary radii.

**Fig. 2.**
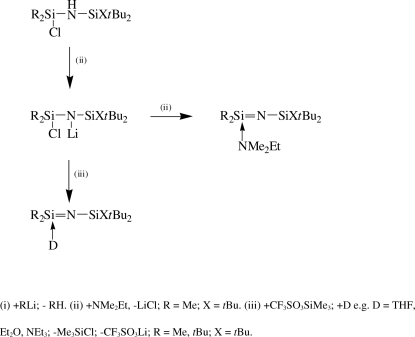
Reaction scheme.

## supplementary crystallographic information

### Comment

We report here the X-ray crystal structure analysis of Ph_3_SiBr. Recently we have synthesized the silanimines Me_2_Si=N-Si*t*Bu_3_ and *t*Bu_2_Si=N-SiX*t*Bu_2_ (*X* = *t*Bu, Cl) as donor adducts (Lerner *et al.*, 2006) (Fig. 2) and the donor-free silanimines *t*Bu_2_Si=N-SiX*t*Bu_2_ (*X* = *t*Bu, Cl, Ph) (Lerner *et al.*, 2001) by salt elimination reactions of Me_2_SiCl-NLi-Si*t*Bu_3_ and *t*Bu_2_SiCl-NM-SiX*t*Bu_2_ (*X* = *t*Bu, Cl, Ph; *M* = Li, Na). However, at room temperature we observed a partial degradation of the donor-free silanimines *t*Bu_2_Si=N-SiX*t*Bu_2_ (*X* = *t*Bu, Cl, Ph) with the formation of isobutene (Lerner *et al.*, 2005). Now we are interested in preparing the phenyl substituted silanimine *t*Bu_2_Si=N-SiPh_3_. In an attempt to synthesize the bromosilylated amine *t*Bu_2_SiBr-NH-SiPh_3_ from *t*Bu_2_SiH-NH-SiPh_3_ and *N*-bromosuccinimide we obtained Ph_3_SiBr as a by-product. X-ray quality crystals of this by-product were grown from a filtrated hexane solution at room temperature.

The title compound, C_18_H_15_BrSi, crystallizes with two almost identical molecules (r.m.s. deviation for all non-H atoms 0.074 Å) in the asymmetric unit. Geometric parameters are in the usual ranges (Bruno *et al.*, 2004). It is isomorphous with chlorotriphenylsilane (Lobkovskii *et al.*, 1981).

### Experimental

A mixture of *t*Bu_2_SiH-NH-SiPh_3_ (0.374 g, 0.90 mmol) and *N*-bromosuccinimide (0.262 g, 1.47 mmol) in 5 ml benzene was stirred for 24 h at room temperature. First the solvent was removed *in vacuo* and then the residue was extracted in hexane. X-ray quality crystals of this by-product were grown from a filtrated hexane solution at room temperature.

### Refinement

H atoms were geometrically positioned and refined using a riding model with fixed individual displacement parameters [U(H) = 1.2 *U*_eq_(C)] and with C—H = 0.95 Å. The final difference map had residual electron density which was located in the center of the phenyl ring C31-C36 and was meaningless.

### Crystal data

**Table:**

C_18_H_15_BrSi	*F*_000_ = 1376
*M**_r_* = 339.30	*D*_x_ = 1.434 Mg m^−^^3^
Monoclinic, *P*2_1_/*c*	Mo *K*α radiation λ = 0.71073 Å
Hall symbol: -P 2ybc	Cell parameters from 32075 reflections
*a* = 18.6306 (13) Å	θ = 2.4–26.3º
*b* = 9.6160 (4) Å	µ = 2.68 mm^−^^1^
*c* = 18.3618 (13) Å	*T* = 173 (2) K
β = 107.174 (5)º	Block, colourless
*V* = 3142.9 (3) Å^3^	0.31 × 0.25 × 0.19 mm
*Z* = 8	

### Data collection

**Table:**

Stoe IPDSII two-circle diffractometer	6141 independent reflections
Radiation source: fine-focus sealed tube	4739 reflections with *I* > 2σ(*I*)
Monochromator: graphite	*R*_int_ = 0.073
*T* = 173(2) K	θ_max_ = 26.1º
ω scans	θ_min_ = 2.3º
Absorption correction: multi-scan(MULABS; Spek, 2003; Blessing, 1995)	*h* = −22→21
*T*_min_ = 0.491, *T*_max_ = 0.630	*k* = 0→11
47324 measured reflections	*l* = 0→22

### Refinement

**Table:**

Refinement on *F*^2^	Secondary atom site location: difference Fourier map
Least-squares matrix: full	Hydrogen site location: inferred from neighbouring sites
*R*[*F*^2^ > 2σ(*F*^2^)] = 0.094	H-atom parameters constrained
*wR*(*F*^2^) = 0.288	*w* = 1/[σ^2^(*F*_o_^2^) + (0.2*P*)^2^] where *P* = (*F*_o_^2^ + 2*F*_c_^2^)/3
*S* = 1.16	(Δ/σ)_max_ < 0.001
6141 reflections	Δρ_max_ = 2.34 e Å^−^^3^
362 parameters	Δρ_min_ = −2.22 e Å^−^^3^
Primary atom site location: structure-invariant direct methods	Extinction correction: none

### Special details

**Table:**

Geometry. All e.s.d.'s (except the e.s.d. in the dihedral angle between two l.s. planes) are estimated using the full covariance matrix. The cell e.s.d.'s are taken into account individually in the estimation of e.s.d.'s in distances, angles and torsion angles; correlations between e.s.d.'s in cell parameters are only used when they are defined by crystal symmetry. An approximate (isotropic) treatment of cell e.s.d.'s is used for estimating e.s.d.'s involving l.s. planes.
Refinement. Refinement of *F*^2^ against ALL reflections. The weighted *R*-factor *wR* and goodness of fit *S* are based on *F*^2^, conventional *R*-factors *R* are based on *F*, with *F* set to zero for negative *F*^2^. The threshold expression of *F*^2^ > σ(*F*^2^) is used only for calculating *R*-factors(gt) *etc*. and is not relevant to the choice of reflections for refinement. *R*-factors based on *F*^2^ are statistically about twice as large as those based on *F*, and *R*- factors based on ALL data will be even larger.

### Fractional atomic coordinates and isotropic or equivalent isotropic displacement parameters (Å^2^)

**Table:**

	*x*	*y*	*z*	*U*_iso_*/*U*_eq_	
Br1	0.40043 (4)	0.50324 (9)	0.36669 (5)	0.0513 (3)	
Si1	0.44440 (9)	0.32247 (18)	0.31491 (10)	0.0287 (4)	
C1	0.4064 (3)	0.3378 (6)	0.2089 (4)	0.0297 (12)	
C2	0.3696 (4)	0.4572 (7)	0.1712 (4)	0.0384 (15)	
H2	0.3634	0.5354	0.2003	0.046*	
C3	0.3425 (4)	0.4625 (9)	0.0928 (5)	0.0472 (18)	
H3	0.3173	0.5436	0.0685	0.057*	
C4	0.3518 (5)	0.3503 (9)	0.0492 (4)	0.051 (2)	
H4	0.3332	0.3542	−0.0048	0.061*	
C5	0.3888 (5)	0.2303 (9)	0.0855 (4)	0.053 (2)	
H5	0.3963	0.1534	0.0560	0.064*	
C6	0.4143 (4)	0.2251 (8)	0.1642 (4)	0.0425 (16)	
H6	0.4378	0.1427	0.1884	0.051*	
C7	0.5489 (3)	0.3357 (6)	0.3495 (4)	0.0304 (13)	
C8	0.5925 (3)	0.3310 (7)	0.2984 (4)	0.0314 (13)	
H8	0.5689	0.3217	0.2452	0.038*	
C9	0.6711 (4)	0.3402 (8)	0.3269 (4)	0.0421 (16)	
H9	0.7004	0.3367	0.2924	0.051*	
C10	0.7064 (4)	0.3543 (8)	0.4039 (4)	0.0426 (16)	
H10	0.7596	0.3606	0.4225	0.051*	
C11	0.6628 (4)	0.3593 (8)	0.4542 (4)	0.0402 (15)	
H11	0.6867	0.3695	0.5073	0.048*	
C12	0.5858 (4)	0.3494 (8)	0.4276 (4)	0.0370 (15)	
H12	0.5573	0.3519	0.4627	0.044*	
C13	0.4096 (3)	0.1588 (7)	0.3471 (4)	0.0312 (13)	
C14	0.3318 (3)	0.1342 (7)	0.3281 (4)	0.0327 (13)	
H14	0.2977	0.2032	0.3012	0.039*	
C15	0.3040 (4)	0.0106 (9)	0.3482 (4)	0.0431 (17)	
H15	0.2513	−0.0048	0.3347	0.052*	
C16	0.3533 (4)	−0.0902 (7)	0.3881 (4)	0.0382 (15)	
H16	0.3354	−0.1754	0.4024	0.046*	
C17	0.4277 (5)	−0.0635 (8)	0.4059 (4)	0.0456 (18)	
H17	0.4614	−0.1331	0.4327	0.055*	
C18	0.4574 (4)	0.0556 (7)	0.3882 (4)	0.0355 (14)	
H18	0.5103	0.0689	0.4036	0.043*	
Br2	0.09145 (4)	−0.23720 (9)	0.11290 (5)	0.0511 (3)	
Si2	0.05044 (9)	−0.06392 (18)	0.17217 (10)	0.0290 (4)	
C19	−0.0543 (3)	−0.0792 (6)	0.1436 (4)	0.0301 (12)	
C20	−0.0925 (3)	−0.0806 (7)	0.1995 (3)	0.0310 (13)	
H20	−0.0650	−0.0729	0.2519	0.037*	
C21	−0.1708 (4)	−0.0932 (8)	0.1780 (4)	0.0419 (16)	
H21	−0.1963	−0.0921	0.2158	0.050*	
C22	−0.2112 (4)	−0.1073 (7)	0.1022 (4)	0.0375 (14)	
H22	−0.2643	−0.1169	0.0879	0.045*	
C23	−0.1737 (4)	−0.1074 (8)	0.0466 (4)	0.0404 (15)	
H23	−0.2014	−0.1183	−0.0056	0.048*	
C24	−0.0975 (4)	−0.0918 (8)	0.0668 (4)	0.0377 (15)	
H24	−0.0731	−0.0894	0.0281	0.045*	
C25	0.0929 (3)	−0.0861 (6)	0.2773 (4)	0.0291 (12)	
C26	0.0895 (4)	0.0262 (7)	0.3249 (4)	0.0371 (14)	
H26	0.0662	0.1106	0.3032	0.044*	
C27	0.1195 (5)	0.0154 (9)	0.4025 (4)	0.0481 (18)	
H27	0.1169	0.0924	0.4341	0.058*	
C28	0.1542 (4)	−0.1087 (9)	0.4358 (4)	0.0438 (17)	
H28	0.1747	−0.1163	0.4896	0.053*	
C29	0.1577 (4)	−0.2189 (8)	0.3892 (4)	0.0414 (16)	
H29	0.1812	−0.3028	0.4113	0.050*	
C30	0.1278 (4)	−0.2103 (7)	0.3101 (4)	0.0373 (14)	
H30	0.1309	−0.2873	0.2788	0.045*	
C31	0.0848 (3)	0.1019 (7)	0.1422 (3)	0.0315 (13)	
C32	0.1619 (4)	0.1250 (8)	0.1564 (4)	0.0355 (14)	
H32	0.1962	0.0530	0.1791	0.043*	
C33	0.1895 (4)	0.2494 (9)	0.1384 (4)	0.0460 (17)	
H33	0.2421	0.2612	0.1482	0.055*	
C34	0.1420 (4)	0.3557 (8)	0.1065 (4)	0.0428 (16)	
H34	0.1617	0.4414	0.0952	0.051*	
C35	0.0630 (4)	0.3379 (8)	0.0904 (4)	0.0444 (17)	
H35	0.0291	0.4106	0.0680	0.053*	
C36	0.0370 (4)	0.2111 (7)	0.1083 (4)	0.0360 (14)	
H36	−0.0156	0.1978	0.0969	0.043*	

### Atomic displacement parameters (Å^2^)

**Table:**

	*U*^11^	*U*^22^	*U*^33^	*U*^12^	*U*^13^	*U*^23^
Br1	0.0467 (5)	0.0495 (5)	0.0644 (5)	−0.0007 (3)	0.0268 (4)	−0.0178 (4)
Si1	0.0234 (8)	0.0314 (9)	0.0324 (8)	−0.0019 (6)	0.0098 (6)	−0.0012 (7)
C1	0.020 (3)	0.028 (3)	0.041 (3)	−0.003 (2)	0.009 (2)	0.001 (3)
C2	0.028 (3)	0.035 (3)	0.051 (4)	0.000 (3)	0.010 (3)	0.009 (3)
C3	0.034 (4)	0.049 (4)	0.052 (4)	−0.006 (3)	0.004 (3)	0.017 (3)
C4	0.054 (4)	0.061 (5)	0.029 (3)	−0.020 (4)	−0.001 (3)	0.014 (3)
C5	0.073 (6)	0.051 (5)	0.034 (4)	−0.015 (4)	0.013 (4)	−0.009 (3)
C6	0.051 (4)	0.035 (4)	0.041 (4)	0.001 (3)	0.013 (3)	0.004 (3)
C7	0.022 (3)	0.032 (3)	0.036 (3)	−0.002 (2)	0.007 (2)	0.002 (2)
C8	0.027 (3)	0.034 (3)	0.035 (3)	−0.004 (2)	0.012 (2)	0.002 (3)
C9	0.033 (3)	0.050 (4)	0.051 (4)	0.000 (3)	0.023 (3)	0.008 (3)
C10	0.029 (3)	0.049 (4)	0.049 (4)	−0.006 (3)	0.010 (3)	0.001 (3)
C11	0.033 (3)	0.044 (4)	0.038 (3)	−0.003 (3)	0.002 (3)	0.002 (3)
C12	0.030 (3)	0.049 (4)	0.033 (3)	−0.008 (3)	0.011 (3)	−0.004 (3)
C13	0.029 (3)	0.032 (3)	0.033 (3)	−0.004 (2)	0.010 (2)	0.004 (2)
C14	0.026 (3)	0.039 (3)	0.033 (3)	−0.002 (3)	0.008 (2)	0.004 (3)
C15	0.030 (3)	0.061 (5)	0.040 (4)	−0.011 (3)	0.012 (3)	0.004 (3)
C16	0.047 (4)	0.029 (3)	0.045 (4)	−0.005 (3)	0.024 (3)	0.005 (3)
C17	0.053 (4)	0.050 (4)	0.046 (4)	−0.024 (4)	0.033 (3)	−0.018 (3)
C18	0.026 (3)	0.041 (4)	0.041 (3)	0.003 (3)	0.013 (3)	0.003 (3)
Br2	0.0459 (5)	0.0498 (5)	0.0634 (5)	0.0038 (3)	0.0250 (4)	−0.0172 (4)
Si2	0.0226 (8)	0.0325 (9)	0.0340 (8)	0.0000 (6)	0.0120 (6)	−0.0026 (7)
C19	0.027 (3)	0.031 (3)	0.034 (3)	−0.001 (2)	0.013 (2)	0.000 (2)
C20	0.027 (3)	0.039 (3)	0.027 (3)	−0.001 (2)	0.009 (2)	0.003 (2)
C21	0.032 (3)	0.057 (4)	0.041 (4)	−0.002 (3)	0.017 (3)	0.007 (3)
C22	0.029 (3)	0.037 (4)	0.047 (4)	−0.002 (3)	0.012 (3)	0.000 (3)
C23	0.037 (3)	0.051 (4)	0.032 (3)	0.002 (3)	0.008 (3)	−0.005 (3)
C24	0.038 (3)	0.045 (4)	0.034 (3)	0.003 (3)	0.017 (3)	−0.006 (3)
C25	0.019 (3)	0.026 (3)	0.043 (3)	0.000 (2)	0.010 (2)	0.005 (2)
C26	0.043 (4)	0.031 (3)	0.037 (3)	0.005 (3)	0.011 (3)	0.006 (3)
C27	0.055 (5)	0.050 (4)	0.039 (4)	−0.013 (4)	0.014 (3)	0.002 (3)
C28	0.037 (4)	0.056 (4)	0.034 (3)	−0.011 (3)	0.003 (3)	0.012 (3)
C29	0.033 (3)	0.040 (4)	0.046 (4)	−0.004 (3)	0.005 (3)	0.013 (3)
C30	0.031 (3)	0.033 (3)	0.047 (4)	0.000 (3)	0.010 (3)	0.004 (3)
C31	0.025 (3)	0.040 (3)	0.031 (3)	−0.003 (3)	0.011 (2)	−0.002 (3)
C32	0.030 (3)	0.047 (4)	0.033 (3)	−0.005 (3)	0.013 (2)	−0.001 (3)
C33	0.042 (4)	0.059 (5)	0.038 (4)	−0.015 (3)	0.014 (3)	0.005 (3)
C34	0.051 (4)	0.043 (4)	0.041 (4)	−0.007 (3)	0.023 (3)	0.005 (3)
C35	0.050 (4)	0.039 (4)	0.048 (4)	0.014 (3)	0.020 (3)	0.014 (3)
C36	0.036 (3)	0.037 (3)	0.039 (3)	0.001 (3)	0.019 (3)	0.001 (3)

### Geometric parameters (Å, °)

**Table:**

Br1—Si1	2.2486 (18)	Br2—Si2	2.2440 (18)
Si1—C13	1.863 (6)	Si2—C31	1.861 (7)
Si1—C7	1.865 (6)	Si2—C25	1.870 (7)
Si1—C1	1.870 (7)	Si2—C19	1.870 (6)
C1—C6	1.393 (10)	C19—C24	1.407 (9)
C1—C2	1.409 (9)	C19—C20	1.412 (8)
C2—C3	1.379 (11)	C20—C21	1.399 (9)
C2—H2	0.9500	C20—H20	0.9500
C3—C4	1.384 (13)	C21—C22	1.379 (10)
C3—H3	0.9500	C21—H21	0.9500
C4—C5	1.406 (12)	C22—C23	1.398 (10)
C4—H4	0.9500	C22—H22	0.9500
C5—C6	1.383 (11)	C23—C24	1.365 (10)
C5—H5	0.9500	C23—H23	0.9500
C6—H6	0.9500	C24—H24	0.9500
C7—C12	1.403 (9)	C25—C26	1.401 (9)
C7—C8	1.412 (9)	C25—C30	1.408 (9)
C8—C9	1.404 (9)	C26—C27	1.373 (10)
C8—H8	0.9500	C26—H26	0.9500
C9—C10	1.379 (11)	C27—C28	1.408 (11)
C9—H9	0.9500	C27—H27	0.9500
C10—C11	1.400 (10)	C28—C29	1.376 (12)
C10—H10	0.9500	C28—H28	0.9500
C11—C12	1.376 (9)	C29—C30	1.395 (10)
C11—H11	0.9500	C29—H29	0.9500
C12—H12	0.9500	C30—H30	0.9500
C13—C18	1.397 (9)	C31—C36	1.398 (9)
C13—C14	1.407 (8)	C31—C32	1.401 (9)
C14—C15	1.389 (10)	C32—C33	1.380 (10)
C14—H14	0.9500	C32—H32	0.9500
C15—C16	1.387 (10)	C33—C34	1.365 (11)
C15—H15	0.9500	C33—H33	0.9500
C16—C17	1.351 (10)	C34—C35	1.424 (11)
C16—H16	0.9500	C34—H34	0.9500
C17—C18	1.353 (10)	C35—C36	1.387 (10)
C17—H17	0.9500	C35—H35	0.9500
C18—H18	0.9500	C36—H36	0.9500
			
C13—Si1—C7	112.1 (3)	C31—Si2—C25	109.0 (3)
C13—Si1—C1	109.7 (3)	C31—Si2—C19	113.9 (3)
C7—Si1—C1	112.6 (3)	C25—Si2—C19	111.6 (3)
C13—Si1—Br1	108.3 (2)	C31—Si2—Br2	107.3 (2)
C7—Si1—Br1	106.1 (2)	C25—Si2—Br2	108.4 (2)
C1—Si1—Br1	107.8 (2)	C19—Si2—Br2	106.3 (2)
C6—C1—C2	117.7 (6)	C24—C19—C20	117.7 (6)
C6—C1—Si1	118.6 (5)	C24—C19—Si2	122.0 (5)
C2—C1—Si1	123.7 (5)	C20—C19—Si2	120.3 (5)
C3—C2—C1	121.2 (7)	C21—C20—C19	120.2 (6)
C3—C2—H2	119.4	C21—C20—H20	119.9
C1—C2—H2	119.4	C19—C20—H20	119.9
C2—C3—C4	120.3 (7)	C22—C21—C20	120.4 (6)
C2—C3—H3	119.8	C22—C21—H21	119.8
C4—C3—H3	119.8	C20—C21—H21	119.8
C3—C4—C5	119.6 (7)	C21—C22—C23	119.7 (6)
C3—C4—H4	120.2	C21—C22—H22	120.2
C5—C4—H4	120.2	C23—C22—H22	120.2
C6—C5—C4	119.6 (8)	C24—C23—C22	120.4 (6)
C6—C5—H5	120.2	C24—C23—H23	119.8
C4—C5—H5	120.2	C22—C23—H23	119.8
C5—C6—C1	121.6 (7)	C23—C24—C19	121.6 (6)
C5—C6—H6	119.2	C23—C24—H24	119.2
C1—C6—H6	119.2	C19—C24—H24	119.2
C12—C7—C8	118.5 (5)	C26—C25—C30	119.1 (6)
C12—C7—Si1	120.2 (5)	C26—C25—Si2	118.2 (5)
C8—C7—Si1	121.4 (5)	C30—C25—Si2	122.6 (5)
C7—C8—C9	119.5 (6)	C27—C26—C25	120.6 (7)
C7—C8—H8	120.2	C27—C26—H26	119.7
C9—C8—H8	120.2	C25—C26—H26	119.7
C10—C9—C8	121.2 (6)	C26—C27—C28	120.6 (7)
C10—C9—H9	119.4	C26—C27—H27	119.7
C8—C9—H9	119.4	C28—C27—H27	119.7
C9—C10—C11	119.1 (6)	C29—C28—C27	118.8 (7)
C9—C10—H10	120.5	C29—C28—H28	120.6
C11—C10—H10	120.5	C27—C28—H28	120.6
C12—C11—C10	120.8 (6)	C28—C29—C30	121.6 (7)
C12—C11—H11	119.6	C28—C29—H29	119.2
C10—C11—H11	119.6	C30—C29—H29	119.2
C11—C12—C7	121.0 (6)	C29—C30—C25	119.2 (7)
C11—C12—H12	119.5	C29—C30—H30	120.4
C7—C12—H12	119.5	C25—C30—H30	120.4
C18—C13—C14	117.4 (6)	C36—C31—C32	116.5 (6)
C18—C13—Si1	123.0 (5)	C36—C31—Si2	123.1 (5)
C14—C13—Si1	119.5 (5)	C32—C31—Si2	120.3 (5)
C15—C14—C13	121.1 (6)	C33—C32—C31	121.8 (7)
C15—C14—H14	119.5	C33—C32—H32	119.1
C13—C14—H14	119.5	C31—C32—H32	119.1
C14—C15—C16	119.8 (6)	C34—C33—C32	120.8 (7)
C14—C15—H15	120.1	C34—C33—H33	119.6
C16—C15—H15	120.1	C32—C33—H33	119.6
C17—C16—C15	117.9 (7)	C33—C34—C35	119.9 (7)
C17—C16—H16	121.1	C33—C34—H34	120.0
C15—C16—H16	121.1	C35—C34—H34	120.0
C16—C17—C18	124.5 (8)	C36—C35—C34	117.9 (6)
C16—C17—H17	117.7	C36—C35—H35	121.1
C18—C17—H17	117.7	C34—C35—H35	121.1
C17—C18—C13	119.3 (7)	C35—C36—C31	123.1 (6)
C17—C18—H18	120.3	C35—C36—H36	118.5
C13—C18—H18	120.3	C31—C36—H36	118.5
			
C13—Si1—C1—C6	−50.7 (6)	C31—Si2—C19—C24	67.8 (6)
C7—Si1—C1—C6	74.9 (6)	C25—Si2—C19—C24	−168.1 (5)
Br1—Si1—C1—C6	−168.4 (5)	Br2—Si2—C19—C24	−50.1 (6)
C13—Si1—C1—C2	129.0 (5)	C31—Si2—C19—C20	−113.1 (5)
C7—Si1—C1—C2	−105.5 (5)	C25—Si2—C19—C20	10.9 (6)
Br1—Si1—C1—C2	11.2 (6)	Br2—Si2—C19—C20	129.0 (5)
C6—C1—C2—C3	−0.1 (10)	C24—C19—C20—C21	−0.4 (10)
Si1—C1—C2—C3	−179.8 (5)	Si2—C19—C20—C21	−179.5 (5)
C1—C2—C3—C4	−0.8 (11)	C19—C20—C21—C22	1.3 (11)
C2—C3—C4—C5	0.2 (12)	C20—C21—C22—C23	−0.7 (11)
C3—C4—C5—C6	1.2 (13)	C21—C22—C23—C24	−0.9 (11)
C4—C5—C6—C1	−2.1 (13)	C22—C23—C24—C19	1.8 (12)
C2—C1—C6—C5	1.6 (11)	C20—C19—C24—C23	−1.2 (10)
Si1—C1—C6—C5	−178.8 (7)	Si2—C19—C24—C23	177.9 (6)
C13—Si1—C7—C12	−66.3 (6)	C31—Si2—C25—C26	49.0 (6)
C1—Si1—C7—C12	169.5 (5)	C19—Si2—C25—C26	−77.8 (5)
Br1—Si1—C7—C12	51.8 (6)	Br2—Si2—C25—C26	165.4 (4)
C13—Si1—C7—C8	113.1 (5)	C31—Si2—C25—C30	−131.0 (5)
C1—Si1—C7—C8	−11.1 (6)	C19—Si2—C25—C30	102.3 (5)
Br1—Si1—C7—C8	−128.9 (5)	Br2—Si2—C25—C30	−14.5 (6)
C12—C7—C8—C9	−0.1 (10)	C30—C25—C26—C27	−0.3 (10)
Si1—C7—C8—C9	−179.5 (5)	Si2—C25—C26—C27	179.8 (6)
C7—C8—C9—C10	−0.2 (11)	C25—C26—C27—C28	−0.1 (11)
C8—C9—C10—C11	0.1 (12)	C26—C27—C28—C29	0.3 (11)
C9—C10—C11—C12	0.4 (12)	C27—C28—C29—C30	−0.2 (11)
C10—C11—C12—C7	−0.7 (12)	C28—C29—C30—C25	−0.2 (10)
C8—C7—C12—C11	0.6 (10)	C26—C25—C30—C29	0.4 (9)
Si1—C7—C12—C11	180.0 (6)	Si2—C25—C30—C29	−179.6 (5)
C7—Si1—C13—C18	−3.8 (7)	C25—Si2—C31—C36	−119.2 (5)
C1—Si1—C13—C18	122.1 (6)	C19—Si2—C31—C36	6.2 (7)
Br1—Si1—C13—C18	−120.5 (5)	Br2—Si2—C31—C36	123.6 (5)
C7—Si1—C13—C14	177.9 (5)	C25—Si2—C31—C32	57.7 (6)
C1—Si1—C13—C14	−56.2 (6)	C19—Si2—C31—C32	−176.9 (5)
Br1—Si1—C13—C14	61.2 (5)	Br2—Si2—C31—C32	−59.4 (5)
C18—C13—C14—C15	−1.3 (10)	C36—C31—C32—C33	0.5 (10)
Si1—C13—C14—C15	177.1 (5)	Si2—C31—C32—C33	−176.7 (5)
C13—C14—C15—C16	0.5 (10)	C31—C32—C33—C34	0.7 (11)
C14—C15—C16—C17	−0.1 (10)	C32—C33—C34—C35	−1.1 (11)
C15—C16—C17—C18	0.7 (11)	C33—C34—C35—C36	0.2 (11)
C16—C17—C18—C13	−1.6 (11)	C34—C35—C36—C31	1.0 (11)
C14—C13—C18—C17	1.9 (10)	C32—C31—C36—C35	−1.3 (10)
Si1—C13—C18—C17	−176.5 (5)	Si2—C31—C36—C35	175.8 (6)
